# From discrete dilated cardiomyopathy to successful cardiac transplantation in congenital disorders of glycosylation due to dolichol kinase deficiency (DK1-CDG)

**DOI:** 10.1007/s10741-012-9302-6

**Published:** 2012-02-11

**Authors:** Livia Kapusta, Nili Zucker, George Frenckel, Benjamin Medalion, Tuvia Ben Gal, Einat Birk, Hanna Mandel, Nadim Nasser, Sarah Morgenstern, Andreas Zuckermann, Dirk J. Lefeber, Arjen de Brouwer, Ron A. Wevers, Avraham Lorber, Eva Morava

**Affiliations:** 1 Children’s Heart Centre, Radboud University Nijmegen Medical Centre, PO Box 9101, 6500 HB Nijmegen, The Netherlands; 2 Pediatric Cardiology Unit, Edith Wolfson Medical Center, Holon, Israel; 3 Heart Institute, Schneider Children’s Medical Centre of Israel, Petach Tikva, Israel; 4 Pediatric Cardiothoracic Surgery, Schneider Children’s Medical Centre of Israel, Petach Tikva, Israel; 5 Department of Cardiothoracic Surgery, Rabin Medical Centre, Petach Tikva, Israel; 6 Department of Cardiology, Rabin Medical Centre, Petach Tikva, Israel; 7 Metabolic Unit, Meyer Children’s Hospital, Rambam Medical Center, Haifa, Israel; 8 Family Physician, High Gallilee-Acre, Israel; 9 Department of Pathology, Rabin Medical Center, Petach Tikva, Israel; 10 Department of Cardiothoracic Surgery, University of Vienna, Vienna, Austria; 11 Institute of Genetic Metabolic and Endocrine Diseases, Department of Neurology, Radboud University Nijmegen Medical Centre, Nijmegen, The Netherlands; 12 Institute of Genetic Metabolic and Endocrine Diseases, Laboratory of Genetic, Endocrine and Metabolic Diseases, Department Laboratory Medicine, Radboud University Nijmegen Medical Centre, Nijmegen, The Netherlands; 13 Department of Human Genetics, Radboud University Nijmegen Medical Centre, Nijmegen, The Netherlands; 14 Pediatric Cardiology and Adults with Congenital Heart Disease, Rambam Medical Center, Haifa, Israel; 15 Institute of Genetic Metabolic and Endocrine Diseases, Department of Pediatrics, Radboud University Nijmegen Medical Centre, Nijmegen, The Netherlands

**Keywords:** Dilated cardiomyopathy, Heart failure, CDG-Im, Congenital disorders of glycosylation, Dolichol kinase deficiency, Cardiac transplantation

## Abstract

Congenital disorders of glycosylation are a growing group of inborn errors of protein glycosylation. Cardiac involvement is frequently observed in the most common form, PMM2-CDG, especially hypertrophic cardiomyopathy. Dilated cardiomyopathy, however, has been only observed in a few CDG subtypes, usually with a lethal outcome. We report on cardiac pathology in nine patients from three unrelated Israeli families, diagnosed with dolichol kinase deficiency, due to novel, homozygous *DK1* gene mutations. The cardiac symptoms varied from discrete, mild dilation to overt heart failure with death. Two children died unexpectedly with acute symptoms of heart failure before the diagnosis of DK1-CDG and heart transplantation could take place. Three other affected children with mild dilated cardiomyopathy at the time of the diagnosis deteriorated rapidly, two of them within days after an acute infection. They all went through successful heart transplantation; one died unexpectedly and 2 others are currently (after 1–5 years) clinically stable. The other 4 children diagnosed with mild dilated cardiomyopathy are doing well on supportive heart failure therapy. In most cases, the cardiac findings dominated the clinical picture, without central nervous system or multisystem involvement, which is unique in CDG syndrome. We suggest to test for DK1-CDG in patients with dilated cardiomyopathy. Patients with discrete cardiomyopathy may remain stable on supportive treatment while others deteriorate rapidly. Our paper is the first comprehensive study on the phenotype of DK1-CDG and the first successful organ transplantation in CDG syndrome.

## Introduction

The process of N-linked protein glycosylation comprises a pathway from the cytoplasm to the endoplasmic reticulum (ER) and the Golgi compartment [1–3]. A recent classification of CDGs distinguishes four major biochemical categories: three involving protein glycosylation (disorders of N-linked glycosylation, O-linked glycosylation, and combined N- and O-glycosylation) and one involving lipid glycosylation [2]. Plasma transferrin isoelectric focusing (TIEF) is used as a simple and reliable biochemical screening tool for CDG [4]. Depending on the localization of a defect, two isofocusing patterns can be distinguished. The so-called type 1 pattern demonstrates increases in even di- and asialotransferrin bands (previously CDG-I). The type 2 pattern shows additional uneven tri- and monosialotransferrin bands (previously CDG-II) [4]. CDG is generally associated with multi-organ symptoms, including psychomotor retardation, ataxia, polyneuropathy, epilepsy, endocrine abnormalities, growth retardation, visual and hearing loss, ichthyosis, cardiac, renal, liver, and gastrointestinal involvement [2, 5–9]. The patients with this syndrome may show a wide clinical variability; however, both hypertrophic [3, 10–15] and/or dilated cardiomyopathy [16–19] have been described.

Dilated cardiomyopathy has been long known to be associated with various inborn errors of metabolism. In many cases, DCM has been associated with abnormal mitochondrial function, like in Kearns-Sayre syndrome (MIM 530000), Acyl-CoA dehydrogenase family member 9 deficiency (MIM 611126), ATP-ase deficiency (MIM 604273), or Barth syndrome (MIM 302060) [20], and also with diverse defects of fatty acid oxidation (Carnitine palmitoyl transferase defect type II; MIM 600649, Long chain Acyl-CoA dehydrogenase defect; MIM 201475, Carnitine deficiency; MIM 212140) or storage disorders (like GM1 gangliosidosis; MIM 230500, haemochromatosis; MIM235200).

Normal glycosylation might also be important for cardiac function. DCM is one of the most important clinical features in DPM3-CDG (MIM 612937) and is observed in tissue-specific types of O-linked mannosylation (Muscular dystrophy-dystroglycanopathy; MIM 607155) [17, 19]. The first description of dilated cardiomyopathy in ALG6-CDG was recently reported in a 9-year-old boy [18]. The child was subsequently placed on ACE inhibitors, and 5 years later, he was weaned off with stabilization of the cardiac function. A lethal form of DCM has been described in DK1-CDG (dolichol kinase defect, MIM 610768; 9q34.11 due to mutations in *TMEM15,* also labeled as the *DK1 and DOLK* gene) [2, 17].

DCM is often diagnosed due to signs and symptoms of advanced heart failure, where cardiac dilatation and impaired contractility are recognized, often in the absence of a recognized etiology. However, initial clinical presentation may be with severe complications: thromboembolism, arrhythmia, or sudden death [21]. Beyond infancy, cardiomyopathy is the most important indication for heart transplantation during childhood. In this work, we report on the cardiac phenotype at presentation and during follow-up of nine DK1-CDG pediatric patients from 3 unrelated families.

## Patients and methods

The diagnosis of dilated cardiomyopathy was made using the standard American and European guidelines for LV size and systolic function in children. In line with these recommendations for standardizing measurements from M-mode echocardiography, it is possible to judge echocardiographic measurements of a particular patient as normal or abnormal using the Z-scores (http://www.parameterz.com, calculate BSA-adjusted Z-scores for common M-Mode measurements; LV end-diastolic and -systolic dimensions, LVEDD and LVESD, respectively). The standard M-mode method of LV systolic function assessment is the shortening fraction (SF). In patients at high risk for diminished function (e.g., SF less than 28%), serial assessments were done. The criteria for the severity of the disease include not only echocardiographic data but also symptoms and function. Mild dilated cardiomyopathy was defined as LV-Z score diameters just above +2SD. Severe dilated cardiomyopathy combined both Z-scores much higher than +2SD with diminished LV (and RV) systolic function, often accompanied by symptoms.

The clinical phenotype of all patients is described in Table 1.

**Table 1:** Clinical phenotype of the nine patients from three unrelated Israeli families diagnosed with DK1-CDG

	I/2	I/5	I/6	II/2	II/3	III/1	III/2	III/3	III/4	GH*	NB*	ASB*	AYB*
Ethnic background/consanguinity	Druze^+^	Druze^+^	Druze^+^	Druze^+^	Druze^+^	Arabic^+^	Arabic^+^	Arabic^+^	Arabic^+^	German	German	Turkish	Turkish
Gender	M	F	F	M	M	F	M	M	M	M	M	M	F
IUGR/low birth weight	–	–	–	–	–	–	–	–	–	–	–	–	–
Muscle hypotonia/motor retardation	–	–	±	–	–	–	–	–	–	+	+	+	+
Neurological abnormalities	–	–	–	–	–	–	–	–	–	+	+	+	+
Visual loss/glaucoma/coloboma	–	–	–	–	–	–	–	–	–	Nystagmus	–	NA	NA
Abnormal MRI of the brain	NA	NA	–	NA	NA	NA	NA	NA	NA	NA	–	NA	NA
Developmental delay	–	–	–	–	–	–	–	–	–	+	+	NA	NA
Dysmorphic features	–	–	±	–	–	–	–	–	–	–	–	–	–
Failure to thrive in infancy	–	–	±	±	–	–	–	–	–	–	+	NA	NA
Delayed puberty	NA	+	+	–	–	+	NA	–	–	NA	NA	NA	NA
Ichthyosis/dry skin/erythroderma	NA	–	+	–	–	±	–	±	±	+	+	+	+
MRHF classification at presentation	Class IV	Class I	Class I	Class I	Class I	Class I	Class IV	Class I	Class I	NA	NA	NA	NA
Dilated cardiomyopathy at presentation^	Severe	Mild	Mild	Mild	Mild	Mild	Severe	Mild	Mild	–	–^#^	+	+
Death (age in years)	9	–	16.5	–	–	–	9	–	–	0.8	0.5	3	Neonate
Heart transplantation (age in years)	–	12	15	11	–	–	–	–	–	–	–	–	–
Genetic defect	Hom. c.1222C > G; (p.His408Asp)			Hom. c.1222C > G; (p.His408Asp)		Hom. c.912G > T; (p.Trp304Cys)				c.295T > A/c.1322A > C; (p.Cys99Ser/p.Tyr441Ser)			

*MRHF* modified ross heart failure, *NA* data not available (not assessed), *M* male, *F* female

* Patient reported by Kranz et al

^#^Cardiomyopathy discovered before death

^+^Consanguinity

^ Echocardiography

### Family I

**Fig. 1:**
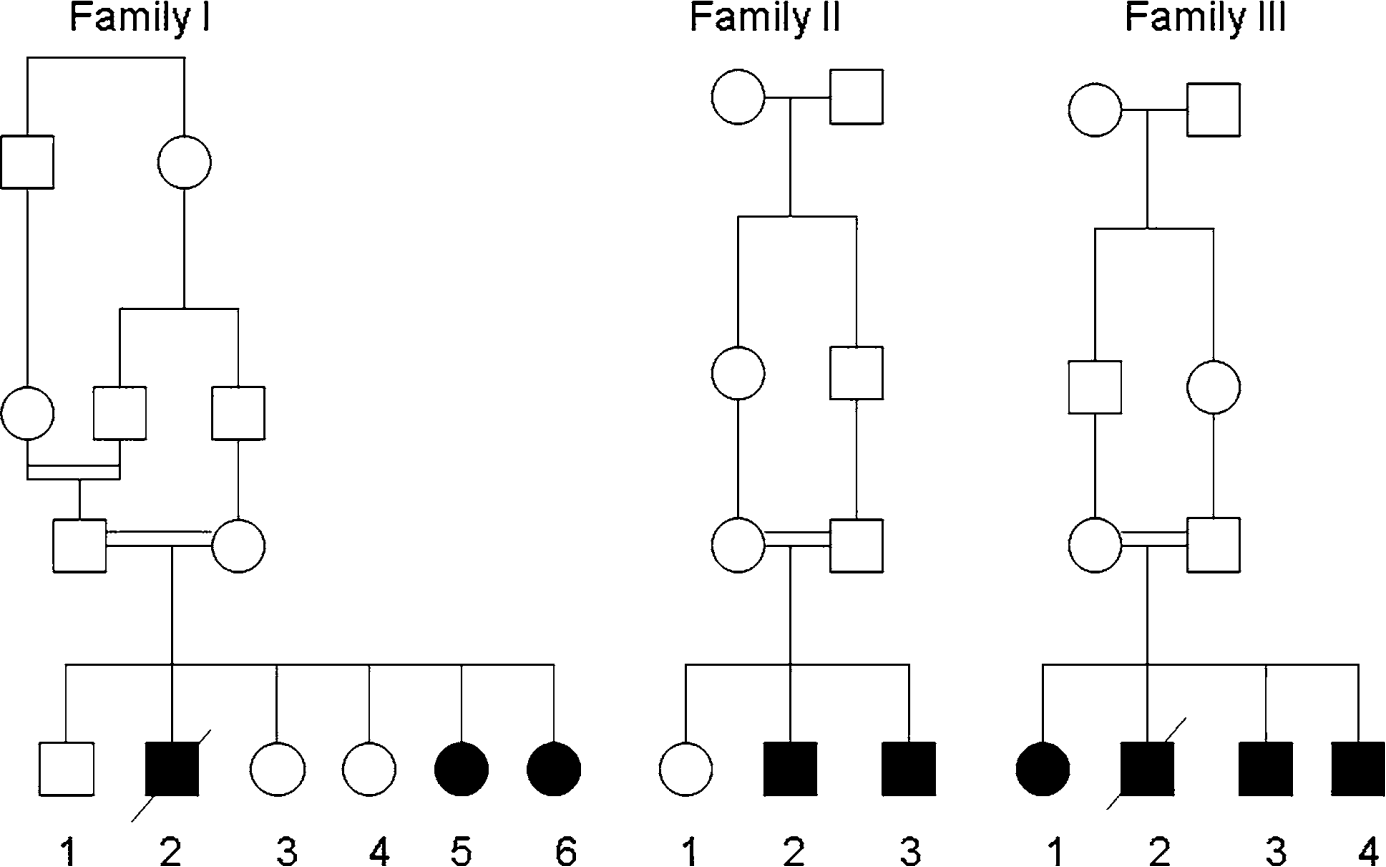
Pedigree of three families with DK1-CDG

*Patient I/2,* the second male child of healthy, consanguineous parents of Druze origin (Fig. 1) was clinically healthy till the age of 9 years when he was acutely admitted with fulminate heart failure due to dilated cardiomyopathy of unknown etiology (LVEDD and LVESD Z-scores unknown). *Patient I/5,* sister of patient I/2, was asymptomatic when diagnosed with a dilated cardiomyopathy at age 7 (LVEDD and LVESD Z-scores 2.18 and 2.58, respectively). *Patient I/6*, a younger sister of patient I/5, had a history of mild hypotonia, failure to thrive, short stature, and ichthyosiform dermatitis. She was diagnosed with mild dilated cardiomyopathy (LVEDD and LVESD Z-scores 2.27 and 2.38, respectively) at the age of 6 without any clinical symptoms.

### Family II

*Patient II/2,* the second male child of healthy, consanguineous parents of Druze origin (and not related to the former Druze family, Fig. 1) was diagnosed with failure to thrive and elevated transaminases during infancy. At 6 years of age, a mild asymptomatic dilatation of the left ventricle was detected on echocardiogram (LVEDD and LVESD Z-scores 2.27 and 2.29, respectively). *Patient II/3,* the younger brother of patient II/2, was clinically asymptomatic and was known with mild hypertransaminasemia. At the age of 5 years, his echocardiography revealed mild dilated cardiomyopathy (LVEDD- and LVESD Z-scores of 2.27 and 2.81, respectively).

### Family III

*Patient III/2,* the second male child of healthy, consanguineous parents of Beduin origin (see also Fig. 1) was known with recurrent episodes of severe neutropenia, microcytic anemia, and frequent infections. At the age of 9 years, he was referred for heart transplantation due to severe heart failure (Class IV modified Ross Heart Failure Classification for Children, LVEDD- and LVESD Z-scores at presentation 3.57 and 7.29, respectively), with repeated life-threatening episodes of rhythm disturbances (e.g., ventricular fibrillation). *Patient III/1*, his older sister, and *Patients III/3* and *III/4*, two younger brothers, were asymptomatic and showed mild to moderate dilated cardiomyopathy on repeated echocardiograms (LVEDD- and LVESD Z-scores at presentation: *patient III/1* 2.06 and 2.29, respectively; *patient III/3* 2.64 and 2.43, respectively; *patient III/4* 2.5 and 1.93, respectively).

### Laboratory investigations

Studies were carried out in all patients alive at the time of the diagnosis of CDG. These included blood cell count, hemoglobin, creatine kinase, liver enzymes, electrolytes, kidney function, prothrombin, activated partial thromboplastin time, cholesterol, triglycerides, thyroid-stimulating hormone, free T4, and albumin level measurements. Additional coagulation studies were performed in most patients (antithrombin 3, factor IX and XI, protein C and S activity).

### Genetic investigations

Based on homozygosity data obtained during routine 250 k SNP array analysis performed in Family I and Family II, and using the results of the biochemical investigations, we selected *DK1* as a candidate gene in our patients. Sequencing of all exons of *DK1 (TMEM15* or *DOLK)* [17] was performed using the ABI PRISM BigDye Terminator Cycle Sequencing V2.0 Ready Reaction Kit and analyzed with the ABI PRISM 3730 DNA analyzer (Applied Biosystems, Foster City, USA) (see Table 2).

**Table 2:** Laboratory values in 9 patients with DK1-CDG

Patients	I/2	I/5	I/6	II/2	II/3	III/1	III/2	III/3	III/4
Microcytic anemia	NA	+	+	±	+	+	+	+	+
Decreased serum iron levels	NA	–	–	+	–	+	NA	+	–
Leucopenia	NA	+	+	+	–	+	NA	+	+
Neutropenia/lymphocytosis	NA	–	–	+	+	+	+	+	+
Thrombopenia	NA	–	–	–	–	–	NA	–	
Elevated liver transaminases	NA	+	+	+	+	–	NA	–	–
Elevated creatine kinase	NA	–	–	–	–	–	NA	–	–
*Abnormal coagulation studies:*									
Decreased Factor IX activity	NA	–	–	–	–	–	NA	–	–
Decreased Factor XI activity	NA	–	+(12%)^$^	+(32%)	+(26%)	+ 34%)	NA	+(17%)	+(28%)
Decreased AT-III activity	NA	+	+	+	+	+	NA		
Decreased protein C activity	NA	+	+(26%)^$^	+(43%)	+(17%)	+(48%)	NA	+(37%)	+(32%)
Prolonged PT	NA	+	+	+	+	+	NA	+	+
Prolonged APTT^+^	NA	–	+	–	+	–	NA	+	+
*Abnormal endocrine studies*									
Cortisol/ACTH	NA	NA	Normal	Low/NA	Normal	Low	NA	Normal	Normal
Growth hormone/IGF1	NA	NA	Normal	Normal	Normal	Normal	NA	Normal	Normal
Insulin	NA	NA	NA	NA	NA	NA	NA	NA	NA
Thyroid function	NA	Normal	Normal	Normal	Normal	Normal	NA	Normal	Normal
*Genetic investigations*									
	Hom. c.1222C > G; (p.His408Asp)			Hom. c.1222C > G; (p.His408Asp)		Hom. c.912G > T; (Trp304Cys)			
Type I transferrin pattern	+	+	+	+	+	+	+	+	+

*NA* data not available (not assessed), *TIEF* transferrin isoelectric focusing

^**+**^Activated partial thromboplastin time

^$^After the transplantation

### Metabolic studies

Plasma samples were collected from EDTA or heparin blood by centrifugation and stored at –20°C. Isoelectric focusing (IEF) of serum transferrin was performed as described [22]. *In Patient II/2,* impaired glycosylation (CDG-Ix) was diagnosed at the age of 4 years [16].

Three children had repeated TIEF analysis upon cardiac transplantation.

Enzyme activity of dolichol kinase was assessed in patient fibroblasts by incubation of C19-dolichol with g32P-CTP. DOLK1 deficiency was confirmed in the fibroblast lines of the index patients from all 3 families.

### Pathological examination

Light microscopy was performed after cardiac tissue was embedded in paraffin. Serial sections of the tissue were evaluated and stained by hematoxylin and eosin, Masson trichrome, Congo red, and phosphotungstic acid hematoxylin. Electronmicroscopic examination was performed in one patient following standard protocols. Thick sections were made of each tissue block and stained with toluidine blue.

## Results

### Family I

*Patient I/2* died after an unsuccessful resuscitation for non-sustained ventricular tachycardia without finding the underlying etiology.

*Patient I/5* developed a slowly progressive cardiac disease. She was treated with oral Carnitine, diuretics, and Digoxin, and later on with Angiotensin Converting Enzyme (ACE) inhibitors and β blockers with no improvement of the cardiac function. Five years later, while waiting for cardiac transplantation for at least 1 year, she exhibited frequent multifocal premature ventricular beats, and later also non-sustained ventricular tachycardia that was treated with Amiodarone. The girl deteriorated rapidly into class IV modified Ross Heart Failure Classification for Children and needed high inotropic cardiac support (e.g., Dobutamine i.v.). Shortly after, at the age of 12, she received heart transplantation and almost 6 years later, she is doing well without episodes of rejection and allograft coronary artery disease.

*Patient I/6* was treated with oral supportive therapy of diuretics, Digoxin, ACE inhibitors and β blockers. At age 10, a transient decrease in the cardiac function was observed during viral infection with a short period of diarrhea, severe neutropenia, and thrombocytopenia (the latter resolved after 10 days). Bone marrow aspiration was normal. At the age of 15 years, following an infection with influenza H1N1, she presented with tachypnoa, diaphoresis at rest, extreme fatigue (class IV modified Ross Heart Failure Classification), and non-sustained ventricular tachycardia treated with Amidarone. Due to rapid deterioration, she was put on ECMO as bridging treatment for successful heart transplantation 6 days later.

Two months later, she presented with rhythm disturbances during viral infection and was treated successfully with Amiodarone and pulse therapy with high dose steroids (Solomedrol i.v.) due to a suspected episode of humoral rejection.

During the first one and a half year after the heart transplantation, she was doing well except for repeated herpetic infections, low weight gain, short stature, and delayed puberty. She then died unexpectedly at home. Permission for autopsy was not given.

### Family II

*Patient II/2* was diagnosed with asymptomatic cardiac dilatation and was treated with low doses ACE inhibitors for 4 years. At age of 11.5, after a short period of upper respiratory tract infection, the boy presented with acute abdominal pain, vomiting, ascites, and peripheral edema. Clinical examination, ECG, and repeated echocardiography demonstrated acute cardiac failure with severe biventricular dilatation of the heart that did not improve with adequate intravenous diuretics, ACE blockers, and high doses inotropics (e.g., Dobutamin and later also Levosimedan i.v.). Few weeks later, he went through heart transplantation.

The post-transplantation pericardial effusion resolved within a few weeks. Twelve months later, he is doing well on maintenance therapy (immunosuppressive medication, low dose steroids, and diuretics). The child is visiting school on regular basis and is playing football (class I modified Ross Heart Failure Classification).

*Patient II/3,* in spite of being asymptomatic with only mild cardiac dilatation, was treated with low doses ACE inhibitors from the age of 5 years. After his brother underwent heart transplantation, β blockers and diuretics were added to his medications in order to try to avoid further cardiac deterioration.

**Fig. 2:**
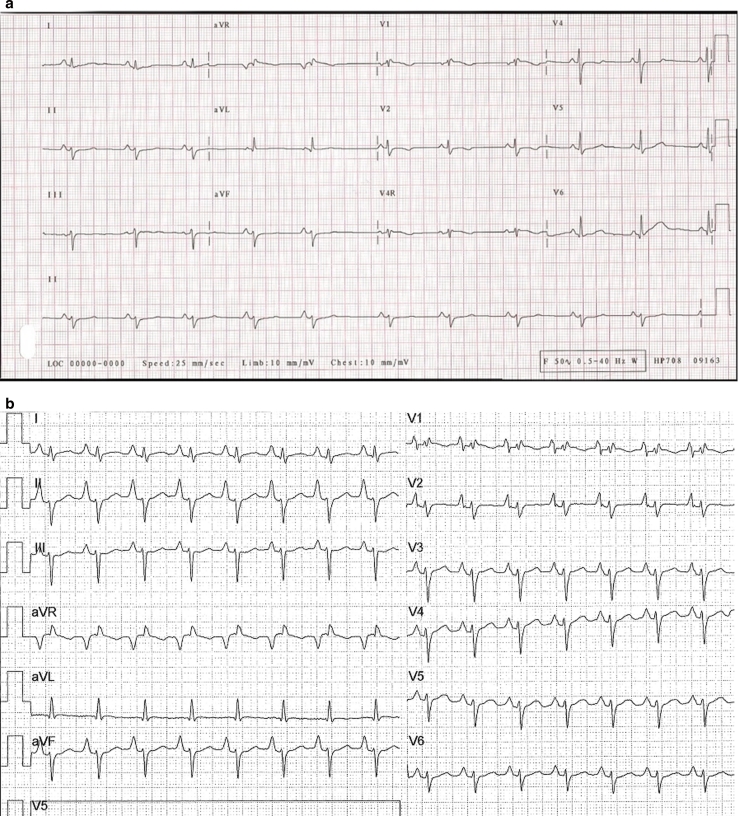
Electrocardiography of a DK1-CDG patient (II/3) with only mild dilation of his LV diameters (**a**) and of his older brother (II/2) just before heart transplantation (**b**)

Five years later on supportive therapy, he is still asymptomatic. His ECG demonstrates normal sinus rhythm, with bifascicular block (incomplete right bundle branch block and left anterior hemi-block), slow upcoming T wave, and QTc of 0.45 s (see Fig. 2a). The ECG was almost identical to the pre-transplantation ECG of his older brother (see Fig. 2b); the latter showing also increased atrial forces (tall p wave). On echocardiography, no further dilatation of his LV diameters has been seen (LV end-diastolic and end-systolic diameters of 44.5 and 30 mm, z-scores of 2.43 and 2.29, respectively).

### Family III

*Patient III/2* presented with high fever, cough, vomiting, peripheral edema and extreme tachypnea, and fatigue (Class IV modified Ross Heart Failure Classification). Upon repeated life-threatening episodes of ventricular fibrillation, he was referred for heart transplantation. He died after unsuccessful resuscitation within 1 month from the first presentation, before a donor heart was available.

*Patient III/1*, his older sister, and *Patients III/3* and *III/4*, two younger brothers, showed asymptomatic (Class I), mild to moderate dilated cardiomyopathy detected by repeated echocardiograms (twice per year). All three children are treated with ACE inhibitors, β blockers, and diuretics; no further deterioration of their cardiac function was observed on repeated echocardiography during the last 5 years of follow-up.

### Laboratory investigations

Most patients presented with chronic microcytic anemia and recurrent episodes of leucopenia without thrombocytopenia. Transaminases were between 80 and 125 U/l (controls <50 U/l) in most individuals (Table 2).

Clotting factor XI and antithrombin III activity were decreased in all patients. Interestingly, no significant bleeding event or thrombotic event was observed in any of the patients.

Endocrine anomalies were mild, including decreased cortisol levels without ACTH elevation and low IGF1 levels. Thyroid function was normal.

### Genetic investigations

Mutation analysis in the *DK1* gene revealed homozygous mutations c.1222C > G; (p.His408Asp) in all affected surviving individuals in *Family I* and *Family II*. The homozygous mutation c.912G>T (p.Trp304Cys) was detected in all affected members of *Family III*.

The mutation c.1222C>G; p.His408Asp in the dolichol kinase gene in family I and family II segregated with the disease: in patients 5 and patient 6 of family I and in patients 2 and 3 of family II, the mutations were detected in a homozygous form, and the parents from both families were proven to be carriers. Homozygous mutations c.912G>T; p.Trp304Cys were confirmed in the affected patients 1, 3, and 4 in family III, whereas their parents were proved to be carriers. The segregation was further supported by showing a carrier state in patients 1 and 4 of family I and patient 1 of family II, correlating with no clinical findings in these healthy family members.

The mutations were screened in a healthy control population of same ethnicity and were absent in 50 healthy control samples. The pathogenicity of the mutations was further corroborated by the high conservation of the mutated amino acids. Both the SIFT and PolyPhen programs predict these changes to be damaging for protein function.

The mutations, which have been found in our patients, are not reported in the Exome Variant Server database [http://evs.gs.washington.edu/EVS/], suggesting that the predicted heterozygosity in the European American and African American population for these mutations is at around 1:10,000.

### Metabolic studies/transferrin isoelectric focusing

The transferrin isoelectric focusing pattern showed a CDG type 1 pattern in all investigated patients. The tetrasialotransferrin (Tf4) ratio was decreased with a significant elevation of the disialotransferrin (Tf2) fraction and increase in the asialotransferrin levels (Tf0). The Tf2 fraction was in the range of 27–38% in family I, in the range of 25–36% in family II, and in the range of 30–38% in family III (controls: 3.3–7.6%).

There was no significant difference between the transferrin pattern before and after transplantation in the investigated individuals’ serum (Table 3).

**Table 3:** Transferrin isoforms before and after transplantation in patients (abnormal levels in bold)

	Patient I/6		Patient II/2		Control range
	Before transplantation	After transplantation	Before transplantation	After transplantation	
Tf0	**10**	**22**	**8**	**9**	0–3.2
Tf1	2	0	1	2	0–5
Tf2	**29**	**38**	**32**	**25**	3.3–7.6
Tf3	8	7	9	10	4.9–10.6
Tf4	**34**	**24**	**31**	**28**	47.3–62.7

Tf4: tetrasialo transferrin, Tf3: trisialotransferrin, Tf2: disialotransferrin, Tf1: monosialotransferrin, Tf0: asialotransferrin

### Pathological examination

*In Patient I/5,* light microscopic evaluation of the affected heart at the time of the heart transplantation revealed dilated cardiomyopathy with moderate interstitial fibrosis and chronic epicarditis.

In *Patient I/6,* biopsy from the affected heart revealed dilated cardiomyopathy with severe interstitial fibrosis.

*In Patient II/2,* biopsy from the affected heart revealed biventricular dilated cardiomyopathy with interstitial fibrosis.

*In Patient III/2,* pathological examination of the affected heart revealed biventricular dilated cardiomyopathy with myocytes fibrosis, severe interstitial fibrosis, chronic epicarditis, and a small endocardial thrombus.

## Discussion

DK1-CDG, also known as dolichol kinase deficiency, is caused by mutations in the *DK1* (also labeled as *TMEM15* or *DOLK*) gene (MIM 610746) [2], which encodes the enzyme responsible for the final step of the de novo biosynthesis of dolichol phosphate. A defect in humans was first described by Kranz et al. [17]. Dolichol phosphate is a lipid carrier embedded in the endoplasmic reticulum (ER) membrane essential for the synthesis of *N*-glycans, GPI-anchors, and protein C- and O-mannosylation. The availability of dolichol phosphate on the cytosolic side of the ER is rate-limiting for *N*-glycosylation, and a defect in its synthesis as in DK1-CDG leads to abnormal N-linked glycosylation [1, 17].

A disruption of the dolichol metabolism leads to a broad spectrum of anomalies. Cantagrel et al. [3] recently described a new CDG form in seven families caused by a defect in polyprenol reductase and affecting dolichol metabolism. In these patients, several different tissues and organs are affected due to *SRD5A3* mutations, such as the cerebellum, diverse ocular structures, or the skin [9]. Cardiac abnormalities were not detected, which was surprising in view of the cardiac involvement found in the corresponding *SRD5A3* mutant animal model [3].

Dolichol is also required for the synthesis of O-mannose-linked glycans, C-mannosylation, and glycophospholipid anchor synthesis. Cardiomyopathy has been detected in O-mannosylation defects [23] and combined N-and O-linked glycosylation defects (DPM3-CDG) [19], giving additional confirmation regarding the interplay of different glycosylation steps and the abnormal glycosylation of diverse proteins in heart, as a consequence. Furthermore, little is known about the glycosylation-independent functions of dolichol, which is a general membrane component in mammalian cells [24, 25].

Very little is known about the clinical outcome of patients with dolichol kinase deficiency. So far, the two families with a lethal form of dolichol kinase deficiency (CDG-Im) described by Kranz et al. [17] are the only patients described in literature. The children died in the first year of life from a severe infection or due to cardiac failure and dilated cardiomyopathy. The neonatally observed neurological involvement and severe muscular hypotonia dominated the clinical presentation. All patients had significant skin findings (hyperkeratosis, ichthyosis, minimal hair growth), and one child had symptoms of hypoglycemia, failure to thrive, and liver involvement.

In the present study, except for laboratory abnormalities, and transient organ involvement during acute infection episodes, no multi-organ involvement was detected. The symptoms of progressive dilated cardiomyopathy were the most prominent in the clinical picture. The skin findings were only sporadic and not so severe. None of the patients had significant dysmorphic features; ophthalmological and neurological assessments were normal; hypoglycemia was not observed; and failure to thrive during infancy was seen in only two patients. Two of our patients presented with signs and symptoms of fulminate heart failure and death, in the beginning of their second decade of life. However, on routine echocardiogram, all affected individuals with the same *TMEM15* (*DK1*) gene mutation showed to have mild to moderate phenotype of dilated cardiomyopathy, already in the first years of life.

The treatment strategies for children with asymptomatic heart failure and cardiomyopathy are, in general, similar to those recommended for adults with heart failure [26, 27]. They include ACE inhibitors and β blockers, in addition to diuretics and even Digoxin. However, the efficacy of the medical treatment in CDG-I patients is completely unknown. Due to acute sudden death of 2 siblings from 2 unrelated families and rapid cardiac deterioration of 3 other patients, a decision was taken to place all children with DK1-CDG, even with mild dilated cardiomyopathy, on ACE inhibitors, β blockers, and diuretics. Rhythm disturbances, e.g., frequent multifocal premature ventricular beats and non-sustained ventricular tachycardia, might be the first sign of cardiac ischemia due to the metabolic status of the myocardium and should be aggressively treated.

Iijima et al. [5] had speculated that the decrease in major coagulation inhibitors, particularly due to impaired glycosylation of AT III, underlies the stoke-like episodes during catabolic states (infection, fever) in CDG syndrome. Arnoux et al. [28] reported on acute vascular events (bleeding and thrombosis) and stroke-like events in patients with CDG-Ia. Thromboses were either venous or arterial. Acute vascular events occurred in patients younger than 15 years, especially with fever and prolonged immobilization. The paradoxical results—abnormal VIII and IX factors in patients without vascular events and normal results in patients with vascular events, while factor XI, antithrombin, protein C, ASAT, and ALAT are abnormal in both groups—could suggest a disequilibrium between prothrombotic and antithrombotic factors in the at-risk group. Vascular events may also occur in patients where glycoproteins are proportionally more hypoglycosylated, particularly protein C. Furthermore, acute fluid loss might potentiate hypercoagulation. Recently, Footitt et al. [13] reported on an 11-year-old girl with a background of learning difficulties and severe dilated cardiomyopathy. Prior to the diagnosis of CDG, her condition deteriorated; she required mechanical support (Excor Berlin Heart) and was listed for cardiac transplant. Despite aggressive therapy, there were persistent difficulties achieving adequate anticoagulation and she developed multiple life-threatening thrombotic complications. In the present study, laboratory hemostatic tests were abnormal in all our DK1-CDG patients. However, none of the patients presented with major bleeding, thrombosis, and stoke-like episodes. Three of our patients were treated successfully with heart transplantation. One needed ECMO while waiting for transplantation. No pre- or postoperative and post-heart catheterization bleeding have been reported in this subgroup. Prophylactic administration of Aspirin and anticoagulants for the prevention of thrombosis in children with dilated cardiomyopathy remains unclear. Bleeding and thromboses have been reported even in the same patient in other subtypes of CDG like in CDG-Ia [28]. Since no acute vascular and transient ischemic “stroke like” were reported in our DK1-CDG patients, prophylaxis with low doses of aspirin can be proposed after a first arterial thrombosis. The families are advised to avoid prolonged immobilization, dehydration, and infection.

We describe a wide spectrum of cardiac involvement in patients with a disorder affecting dolichol synthesis. In contrast to the severe outcome with early death reported by Kranz et al. [17], we experienced various clinical cardiac outcome. In our 3 families, siblings did not show any symptoms of cardiac failure in infancy and early childhood. Cardiac evaluation took place only after the death of a first child (family I and III). So discrete dilated cardiomyopathy in early infancy and childhood cannot be ruled out.

This is the first comprehensive description of clinical, histological, and laboratory findings in a series of patients with dolichol kinase defect. DK1-CDG is a disorder with dilated cardiomyopathy with ichthyosiform skin abnormalities and hypotonia in some of the patients with a highly variable outcome. We expand the clinical phenotype with the observation of microcytic anemia and report on the laboratory abnormalities of abnormal coagulation and normal endocrine studies in dolichol kinase defect.

There are several DK1-CDG patients in our cohort with long-term survival. In the presence of severe coagulation abnormalities and decreased antithrombic factors, the patients had no significant bleeding episodes and three of them underwent successful heart transplantation without thrombotic events. Given the high variability of the clinical phenotype in DK1-CDG, it is important to realize that life-threatening dilated cardiomyopathy can be the single clinical manifestation of congenital disorders of glycosylation. Once the diagnosis of DK1-CDG is established, repeated echocardiograms, at least twice per year, are indicated. Treatment for heart failure should be well thought of even in asymptomatic DK1-CDG patients with discrete dilated cardiomyopathy. Our paper is the first comprehensive study on the phenotype of DK1-CDG and the first successful organ transplantation in CDG syndrome.

## References

[1] Freeze HH (2009). Update on congenital disorders of glycosylation. Glycobiology.

[2] Jaeken J, Hennet T, Matthijs G, Freeze HH (2009). CDG nomenclature: Time for a change!. Biochimica et Biophysica Acta-Molecular Basis of Disease.

[3] Cantagrel V, Lefeber DJ, Ng BG, Guan Z, Silhavy JL, Bielas SL (2010). SRD5A3 is required for converting polyprenol to dolichol and is mutated in a congenital glycosylation disorder. Cell.

[4] Wopereis S, Lefeber DJ, Morava E, Wevers RA (2006). Mechanisms in protein *O*-glycan biosynthesis and clinical and molecular aspects of protein *O*-glycan biosynthesis defects: a review. Clin Chem.

[5] Iijima K, Murakami F, Nakamura K, Ikawa S, Yuasa I, Motosumi H (1994). Hemostatic studies in patients with carbohydrate-deficient glycoprotein syndrome. Thromb Res.

[6] Eklund EA, Sun L, Westphal V, Northrop JL, Freeze HH, Scaglia F (2005). Congenital disorder of glycosylation (CDG)-Ih patient with a severe hepato-intestinal phenotype and evolving central nervous system pathology. J Pediatr.

[7] Truin G, Guillard M, Lefeber D, Sykut-Cegielska J, Adarnowicz M, Hoppenreijs E (2008). Pericardial and abdominal fluid accumulation in congenital disorder of glycosylation type Ia. Mol Genet Metab.

[8] Morava E, Wosik HN, Sykut-Cegielska J, Adamowicz M, Guillard M, Wevers RA (2009). Ophthalmological abnormalities in children with congenital disorders of glycosylation type I. Br J Ophthalmol.

[9] Morava E, Wevers RA, Cantagrel V, Hoefsloot LH, Al-Gazali L, Schoots J (2010). A novel cerebello-ocular syndrome with abnormal glycosylation due to abnormalities in dolichol metabolism. Brain.

[10] Clayton PT, Winchester BG, Keir G (1992). Hypertrophic obstructive cardiomyopathy in a neonate with the carbohydrate-deficient glycoprotein syndrome. J Inherit Metab Dis.

[11] Garcia Silva MT, de CJ, Stibler H, Simon R, Chasco YA, Mateos F (1996). Prenatal hypertrophic cardiomyopathy and pericardial effusion in carbohydrate-deficient glycoprotein syndrome. J Inherit Metab Dis.

[12] Malhotra A, Pateman A, Chalmers R, Coman D, Menahem S (2009). Prenatal cardiac ultrasound finding in congenital disorder of glycosylation type 1a. Fetal Diagn Ther.

[13] Footitt EJ, Karimova A, Burch M, Yayeh T, Dupre T, Vuillaumier-Barrot S et al. (2009) Cardiomyopathy in the congenital disorders of glycosylation (CDG): a case of late presentation and literature review. J Inherit Metab Dis 10.1007/s10545-009-1262-1 19757145

[14] Marquardt T, Hulskamp G, Gehrmann J, Debus V, Harms E, Kehl HG (2002). Severe transient myocardial ischaemia caused by hypertrophic cardiomyopathy in a patient with congenital disorder of glycosylation type Ia. Eur J Pediatr.

[15] Kranz C, Basinger AA, Gucsavas-Calikoglu M, Sun LW, Powell CM, Henderson FW (2007). Expanding spectrum of congenital disorder of glycosylation Ig (CDG-Ig): sibs with a unique skeletal dysplasia, hypogammaglobulinemia, cardiomyopathy, genital malformations, and early lethality. Am J Med Genetics A.

[16] Iancu TC, Mahajnah M, Manov I, Cherurg S, Knopf C, Mandel H (2007). The liver in congenital disorders of glycosylation: ultrastructural features. Ultrastruct Pathol.

[17] Kranz C, Jungeblut C, Denecke J, Erlekotte A, Sohlbach C, Debus V (2007). A defect in dolichol phosphate biosynthesis causes a new inherited disorder with death in early infancy. Am J Hum Genet.

[18] Al-Owain M, Mohamed S, Kaya N, Zagal A, Matthijs G, Jaeken J (2010) A novel mutation and first report of dilated cardiomyopathy in ALG6-CDG (CDG-Ic): a case report. Orphanet J Rare Dis 5 10.1186/1750-1172-5-7 PMC2861021 20398363

[19] Lefeber DJ, Schonberger J, Morava E, Guillard M, Huyben KM, Verriip K (2009). Deficiency of Dol-P-man synthase subunit DPM3 bridges the congenital disorders of glycosylation with the dystroglycanopathies. Am J Hum Genet.

[20] Wortmann SB, Rodenburg RJT, Jonckheere A, de Vries MC, Huizing M, Heldt K (2009). Biochemical and genetic analysis of 3-methylglutaconic aciduria type IV: a diagnostic strategy. Brain.

[21] Baig MK, Goldman JH, Caforio AL, Coonar AS, Keeling PJ, McKenna WJ (1998). Familial dilated cardiomyopathy: cardiac abnormalities are common in asymptomatic relatives and may represent early disease. J Am Coll Cardiol.

[22] Wopereis S, Grunewald S, Morava E, Penzien JM, Briones P, Garcia-Silva MT (2003). Apolipoprotein C-III isofocusing in the diagnosis of genetic defects in *O*-glycan biosynthesis. Clin Chem.

[23] van Reeuwijk J, Grewal PK, Salih MAM, de Bernabe DBV, McLaughlan JM, Michielse CB (2007). Intragenic deletion in the LARGE gene causes Walker-Warburg syndrome. Hum Genet.

[24] Rip JW, Rupar CA, Ravi K, Carroll KK (1985). Distribution, metabolism and function of dolichol and polyprenols. Prog Lipid Res.

[25] Cantagrel V, Lefeber DJ (2011) From glycosylation disorders to dolichol biosynthesis defects: a new class of metabolic diseases. J Inherit Metab Dis 859–867 10.1007/s10545-011-9301-0 PMC3137772 21384228

[26] Kantor PF, Mertens LL (2010). Clinical practice: heart failure in children. Part II: current maintenance therapy and new therapeutic approaches. Eur J Pediatr.

[27] Silva JN, Canter CE (2010). Current management of pediatric dilated cardiomyopathy. Curr Opin Cardiol.

[28] Arnoux JB, Boddaert N, Valayannopoulos V, Romano S, Bahi-Buisson N, Desguerre I (2008). Risk assessment of acute vascular events in congenital disorder of glycosylation type Ia. Mol Genet Metab.

